# Cystic Lung Changes in Down Syndrome: A Case Report

**DOI:** 10.7759/cureus.31867

**Published:** 2022-11-24

**Authors:** Fuad H Alshaibani

**Affiliations:** 1 Radiology, Salmaniya Medical Complex, Manama, BHR

**Keywords:** cystic lung changes, case report, pneumonia, computed tomography, lung cysts, down syndrome

## Abstract

A wide spectrum of multi-organ complications have been associated with Down syndrome. Pulmonary complications are a leading cause of morbidity and mortality in Down syndrome. A four-year-old boy with Down syndrome presented to our emergency department with a cough and shortness of breath. He had signs of respiratory distress and decreased air entry in the right lung. A chest radiograph revealed airspace opacity and an air bronchogram in the right lung, both consistent with pneumonia. Oxygen saturation was not maintained on a non-rebreather mask, and the patient required admission to the intensive care unit, where he underwent intubation and mechanical ventilation. With the aggressive antibiotic therapy, the patient had improvements in terms of laboratory and radiographic findings. However, clinical symptoms persisted. Hence, a computed tomography (CT) scan was performed, which demonstrated findings of pulmonary edema and unexpected findings of subpleural cystic lung changes bilaterally with significant replacement of the right middle lobe with these cysts. Initially, these cysts caused significant confusion for the treating physicians and were misinterpreted as honeycombing changes related to end-stage lung disease. However, radiologists confirmed the incidental nature of these cysts in patients with Down syndrome. Appropriate recognition of this entity is crucial to avoid its misinterpretation, which may cause unnecessary laboratory and radiological investigations.

## Introduction

Down syndrome is one of the most well-recognized genetic disorders worldwide. It affects one in every 700 live births, making it the most common chromosomal abnormality in live births [1]. A wide range of complications in all organ systems have been associated with Down syndrome. Cardiac and pulmonary conditions are the leading cause of morbidity and mortality in Down syndrome. The pulmonary complications involve the entire lung, from the airways to the lower respiratory tract and pulmonary vasculature [2]. Previous research indicated that respiratory complications were the most common cause of hospital admission among patients with Down syndrome [1]. Additionally, other conditions associated with Down syndrome, such as hypotonia, may contribute to or result in respiratory disease. Common pulmonary complications of Down syndrome include recurrent respiratory infections, sleep disorders, laryngomalacia, tracheobronchomalacia, and pulmonary hypertension [2]. Here, I report the case of a child with Down syndrome who was admitted with pneumonia complicated by septic shock, requiring intensive care unit admission. On a computed tomography (CT) scan, the child was found to have extensive cystic changes that initially caused significant confusion, and the findings were misinterpreted as honeycombing related to end-stage lung disease. Awareness of the limited clinical significance of these cystic changes is crucial to avoid unnecessary investigations [3].

## Case presentation

A four-year-old boy with Down syndrome presented to our emergency department with a one-day history of coughing and decreased feeding. The mother reported that the cough had been increasing in severity and had started to cause shortness of breath. She also reported a history of decreased feeding during the current illness. The child has a history of sick contact with his sister, who has just recovered from an upper respiratory tract infection. There was no history of fever, vomiting, or changes in bowel habits. The child was up-to-date on the vaccination schedule. The medical history was remarkable for severe pneumonia one year ago, for which the patient was admitted to the intensive care unit for 25 days and required endotracheal intubation and mechanical ventilation. The child had a history of congenital heart defects in the form of atrioventricular septal defects and patent ductus arteriosus that underwent successful surgical closure. The prenatal history was unremarkable.

On examination, the child appeared agitated and had respiratory distress evident in severe chest retractions. Vital signs revealed a normal temperature of 36.8 °C, tachycardia with a heart rate of 175 beats per minute, tachypnea with a respiratory rate of 52 breaths per minute, blood pressure of 83/48 mmHg, and oxygen saturation of 83% in room air. Respiratory examination revealed generalized decreased air entry on the right lung, along with bronchial breathing and crepitations.

**Figure 1:**
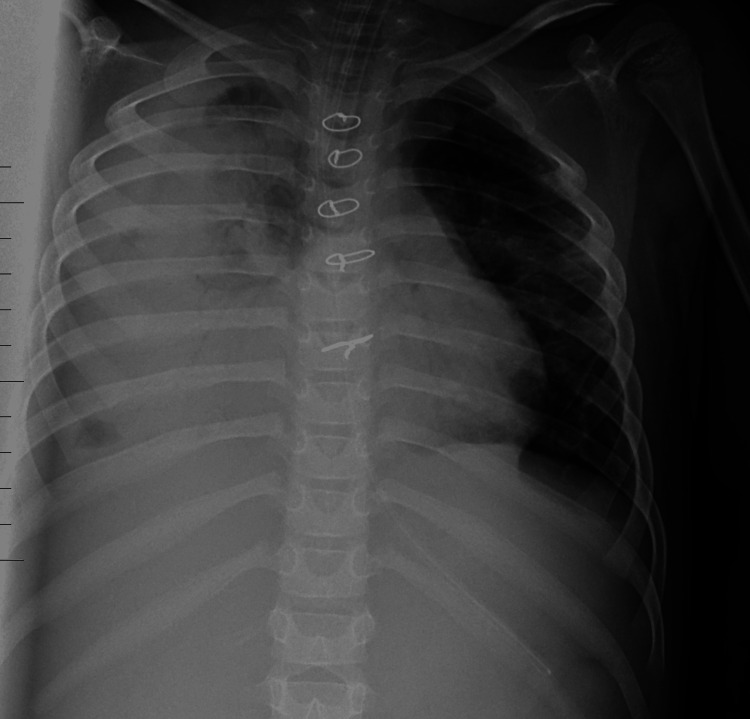
A frontal chest radiograph shows near complete opacification of the right hemithorax with the presence of an air bronchogram, consistent with extensive pneumonia. Sternotomy wires from previous cardiac surgery are seen.

Initial laboratory investigation of hematological parameters revealed leukocytosis (16,300/μL), a hemoglobin level of 11.5 g/dL, and a platelet count of 213,000/μL. The urea and electrolyte levels were within the normal range. The arterial blood gas analysis showed metabolic acidosis with a pH of 7.2 and a base deficit of 6.9. The frontal chest radiograph revealed extensive airspace opacity with an air bronchogram involving most of the right hemithorax (Figure 1).

**Figure 2:**
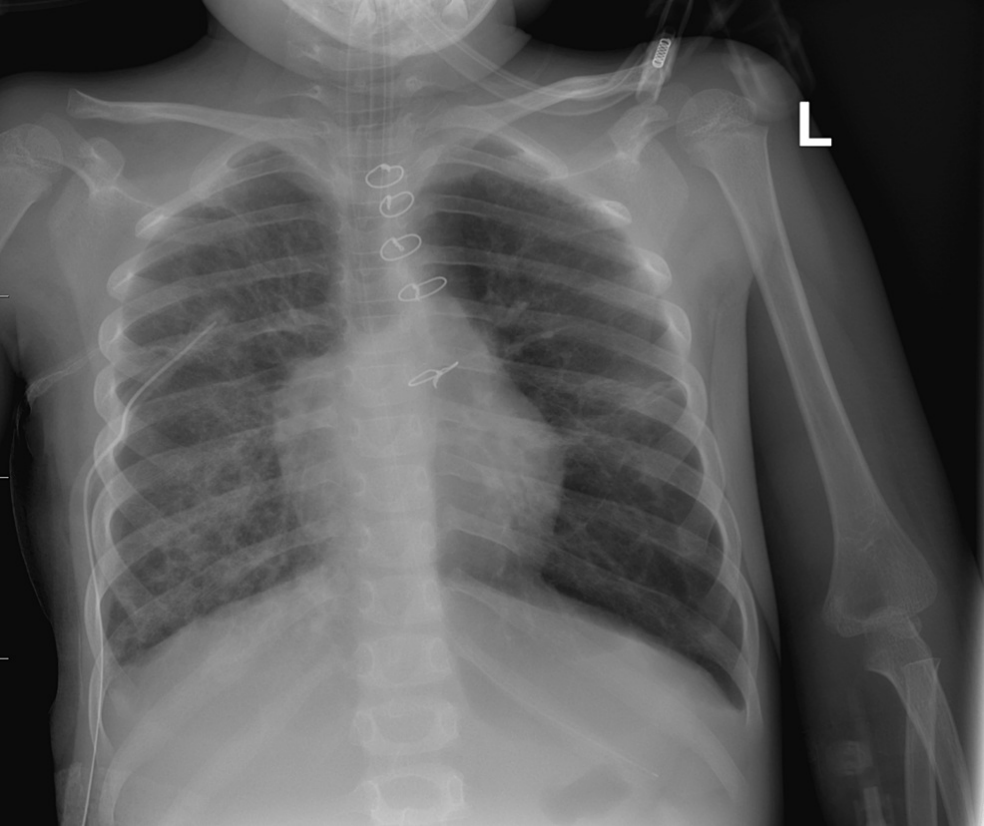
The frontal chest radiograph shows remarkable interval regression in the previously seen consolidation of the right hemithorax.

The patient was then admitted with a clinical impression of severe pneumonia. The patient received intravenous fluid rehydration. Intravenous antibiotic therapy was administered in the form of ceftriaxone and clindamycin. The patient was kept on a 10-liter non-rebreather oxygen mask, and the oxygen saturation reached 92%-94%. Despite these measures, oxygen saturation was not maintained. Therefore, the patient was kept on a high-flow nasal cannula and transferred to the intensive care unit, where he underwent endotracheal intubation and mechanical ventilation. The patient’s condition deteriorated, and he developed multiorgan failure, including hypotension, acute kidney injury, and coagulopathy. Antibiotic therapy was changed to intravenous linezolid and piperacillin/tazobactam. A chest tube was inserted, and 150 ml of yellowish fluid was drained. The fluid had a leukocyte count of 3400 cells/μL (90% neutrophils), lactate dehydrogenase of 300 U/L, and albumin of 2.1 g/dL. The culture revealed positive growth for Streptococcus pneumoniae. Subsequently, a gradual improvement in the patient’s condition was observed, and a successful extubation was performed. Inflammatory markers and chest radiographs also showed improvement over the course of the stay (Figure 2).

**Figure 3:**
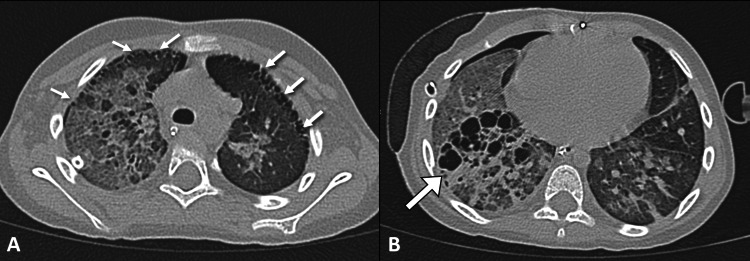
An axial CT scan of the chest at the level of the upper (A) and middle thorax (B) shows ground-glass opacity with interlobular septal thickening related to pulmonary edema along with subpleural cysts bilaterally (small arrows). The right middle lobe is significantly replaced by variable-sized cysts (long arrow). A small pneumothorax (related to chest tube insertion) is noted. CT: computed tomography

Despite the improvement in the radiological and laboratory parameters, the patient experienced an increased oxygen requirement. The patient underwent chest computed tomography to rule out necrotizing pneumonia or pleural effusion. The scan demonstrated changes in cardiac pulmonary edema evident by ground-glass opacities, with the predominant perihilar distribution associated with interlobular septal thickening and bronchial wall thickening. Subpleural cysts were observed bilaterally in both the upper and lower lobes. The right middle lobe was significantly replaced by variable-sized cystic spaces that extended along the bronchovascular bundle (Figure 3). At first glance, the cystic changes in the lungs were striking and a source of confusion about their possible etiology. However, the radiologist reassured the physicians that these changes can be an incidental finding in patients with Down syndrome. The patient underwent fluid restriction with strict input/output fluid balance. The child was discharged in good condition after a 30-day hospital stay.

## Discussion

I present a case of a child with Down syndrome who was admitted to the hospital with severe pneumonia and was found to have extensive lung changes that initially confused the treating physicians about their exact nature and etiology. The first description of the cystic lung changes in Down syndrome was made by Joshi et al. [4] in 1986, on the postmortem examination of two young infants with Down syndrome and congenital heart defects. They proposed a number of explanations for these cystic changes, including the consequences of cardiac chamber enlargement due to underlying cardiac defects as well as pulmonary hypoplasia [4].

Limited reports have described the cystic lung changes in Down syndrome [5-7]. Such cystic changes have a subpleural distribution. Gonzalez et al. [6] performed an autopsy study that involved 98 infants with Down syndrome, including 89 live-born and nine stillborn, and found a prevalence of 20% for subpleural cystic lung changes. Interestingly, they reported that such lung changes were only found in two patients out of 8000 pediatric postmortem examinations of patients without Down syndrome. Furthermore, stillborn patients with Down syndrome did not have subpleural cysts on postmortem examination [6]. This was consistent with the postulation that reduced alveolar production occurs later in postnatal life.

Biko et al. [7] conducted a retrospective study to estimate the prevalence of cystic lung changes on computed tomography scans in patients with Down syndrome, which revealed that 36% of patients with Down syndrome had subpleural cysts. Some previous studies indicated an association between subpleural cysts, congenital heart defects, and a higher rate of mortality [5,6]. However, these findings were not confirmed by later studies. In particular, they found no significant association between cystic changes and the presence of congenital heart defects, the use of extracorporeal membrane oxygenation, or the need for prolonged mechanical ventilation. However, there was a significant association with prematurity.

## Conclusions

Patients with Down syndrome may have cystic lung changes with subpleural and intraparenchymal distribution on computed tomography. Pediatric physicians and radiologists need to be aware of this entity in patients with Down syndrome. Appropriate recognition is crucial to avoid misinterpretation, which may cause unnecessary interventions. Future studies are needed to investigate the long-term effects of these cystic changes on patients.

## References

[1] Day SM, Strauss DJ, Shavelle RM, Reynolds RJ (2005). Mortality and causes of death in persons with Down syndrome in California. Dev Med Child Neurol.

[2] McDowell KM, Craven DI (2011). Pulmonary complications of Down syndrome during childhood. J Pediatr.

[3] Manuel DA, Irodi A, Sudhakar SV, Varkki S (2016). Abnormal chest radiograph due to a common lung finding in Down syndrome. Oman Med J.

[4] Joshi VV, Kasznica J, Ali Khan MA, Amato JJ, Levine OR (1986). Cystic lung disease in Down's syndrome: a report of two cases. Pediatr Pathol.

[5] George M, Amodio J, Lee H (2016). Cystic lung disease in Down syndrome: a case report and literature review. Case Rep Pediatr.

[6] Gonzalez OR, Gomez IG, Recalde AL, Landing BH (1991). Postnatal development of the cystic lung lesion of Down syndrome: suggestion that the cause is reduced formation of peripheral air spaces. Pediatr Pathol.

[7] Biko DM, Schwartz M, Anupindi SA, Altes TA (2008). Subpleural lung cysts in Down syndrome: prevalence and association with coexisting diagnoses. Pediatr Radiol.

